# Successful prioritisation of inguinal herniotomies in children during the COVID-19 pandemic to minimise emergency presentations

**DOI:** 10.1186/s43159-023-00243-1

**Published:** 2023-05-01

**Authors:** Mahmoud Marei Marei, Ahmed Sobhy Hassan, Mohamed Kamel, Aiden Moore, Olugbenga Michael Aworanti

**Affiliations:** 1 Manchester University NHS Foundation Trust, Royal Manchester Children’s Hospital, Oxford Road, Manchester, M13 9WL UK; 2 Faculty of Medicine (Kasr Alainy), Cairo University Hospitals, Cairo University, Cairo, 11562 Egypt; 3 Birmingham Women’s and Children’s NHS Foundation Trust, Birmingham Children’s Hospital, Steelhouse Lane, Birmingham, B4 6NH UK

**Keywords:** COVID-19, Surgical prioritisation, NHS waiting list, Congenital inguinal hernia, Pediatric hernia, Elective herniotomy, Incarcerated hernia, Emergency herniotomy

## Abstract

**Background:**

The coronavirus disease 2019 (COVID-19) disrupted the delivery of elective surgery in children. We introduced guidance to mitigate this impact. By reviewing the outcomes for inguinal herniotomies, we aimed to determine if this guidance has enabled us to prevent an increase in the elective surgery wait time and therefore the need for emergency surgery for incarcerated hernias. This report aims to share our learnt lessons about responding to a crisis limiting accessibility to elective surgery.

**Results:**

We performed a retrospective review of all elective and emergency herniotomies performed between April 1 and September 30, 2019 (pre-COVID-19) and the same period in 2020 (post-COVID-19). We compared the data on wait time from referral to clinic review/elective surgery and incarceration rates. During the study period in 2019, 76 elective herniotomies were performed compared to 46 in 2020. We did not observe a simultaneous increase in emergency herniotomies in 2020 (27 [2020] vs 25 [2019], OR [95% CI] = 1.53 [0.79–2.9]; *p* = 0.2). The median time from referral to elective surgery in 2019 compared to 2020 did not differ (56 vs 59 days, respectively; *p* = 0.61). In 2020, 72% of children that required emergency surgery had not been previously referred to our service and the median age (interquartile range) at which they presented with an incarcerated hernia was 2.8 months (2.1–13.7 months).

**Conclusion:**

By adhering to local guidelines for resumption of elective activity, the pandemic did not result in children waiting longer to be seen by a surgeon for a suspected inguinal hernia. As a result, we did not perform more emergency herniotomies. Urgent prioritisation of hernias in infants, from birth up to 3 months old, was a beneficial strategy. Public health education on childhood hernias will improve outcomes.

**Supplementary Information:**

The online version contains supplementary material available at 10.1186/s43159-023-00243-1.

## Background

The coronavirus disease 2019 (COVID-19) caused by the severe acute respiratory syndrome coronavirus 2 (SARS-CoV-2) continues to affect populations worldwide. It has been established that children suffer less morbidity and mortality [1]; however, the indirect effects of the pandemic remain considerable. Particularly, the delivery of elective surgery in children has been disrupted as healthcare systems became overwhelmed, national lockdowns were introduced, and concerns arose on how to safely deliver elective surgery during the pandemic. The medical society quickly recognised the significance of this disruption to the delivery of acute and routine healthcare services during the pandemic [2, 3], and several medical bodies developed guidelines on how to mitigate this impact and resume care for non-COVID-19-related illnesses [1, 4–8].

In the UK, national lockdown was first introduced from the 16^th^ of March 2020. The initial response by our institution was to cease all elective surgical activity from the 17^th^ of March 2020 and all outpatient department (OPD) activity from 26^th^ of March 2020, until a strategy to safely deliver these services was devised. Subsequently, the Federation of Surgical Specialty Associations (FSSA) [4], the National Institute for Health and Care Excellence (NICE) [6], and the Royal College of Paediatrics and Child Health (RCPCH) [5] published guidance to safely deliver elective care to children, including surgeries. Using this guidance our institution developed local guidelines, in line with it, to underpin the resumption of elective activity.

By reviewing our data on inguinal herniotomies, we aimed to determine if this guidance enabled us to prevent an increase in the wait time between either a child’s referral was received, or the child was seen in the OPD and elective herniotomy was performed. If wait times were prolonged, we sought to determine if this resulted in fewer elective surgeries and an increase in the number of emergency herniotomies performed for incarcerated hernias. Finally, we determined what age groups required emergency herniotomy during the pandemic and how this age group compared to the age group prioritisation guidance.

## Methods

We performed a retrospective review of the records of all inguinal herniotomies performed at our institution between April 1 to September 30, 2019 (pre-COVID-19) and between April 1 to September 30, 2020 (post-COVID-19).

Within these time periods, we analysed two groups; those that were scheduled electively for their surgery and those that required emergency surgery within 48 h of presentation because of an incarcerated inguinal hernia. We analysed the demographic data and wait time from hernia referral received/OPD review to hernia surgery.

These operations were either via an open groin incision or by a laparoscopic approach; when done laparoscopically, a contralateral open deep internal ring was repaired, if present.

We compared the incarceration rates between both time periods (2019 pre-COVID-19 and 2020 post-COVID-19). Furthermore, we compared our incarceration rates (defined as children seen and diagnosed as having an inguinal hernia in the OPD, and while awaiting elective surgery presenting with an incarcerated hernia, which required emergency surgery on that admission) to the published rates.

Our institution is a large National Health Service (NHS) foundation trust that operates the “hub-and-spoke” model of health care delivery, whereby more complex procedures are performed at the main site while basic services are delegated to secondary sites. This study was performed at our main site for both time periods.

The Federation of Surgical Specialty Associations (FSSA) issued the guidance: Clinical Guide to Surgical Prioritisation During the Coronavirus Pandemic  [4], which designates inguinal hernia under 3 months of age as Priority 2—to be performed in < 1 months; inguinal hernia 3–12 months of age as Priority 3—to be performed in < 3 months; and inguinal hernia (> 12 months of age) as Priority 4—to be performed in > 3 months. We tailored our OPD activity, scheduling process and theatre procedures to comply with this guidance.

The Fisher’s exact test was used to analyse categorical variables, and Mann–Whitney *U* test was used to analyse non-parametric continuous data. Significance was set at *p* < 0.05.

## Results

To provide context to the results presented below, encompassing our main and secondary sites, the October 2018 Getting It Right First Time (GIRFT) data, reported that between April 2016 and March 2018 (23 months), 880 herniotomies (15% bilateral) were performed at Manchester University NHS Foundation Trust. This was the largest number of cases performed for that period compared to other NHS Trusts in the UK [9].

Between April 1 and September 30th, 2019 (pre-COVID-19) and 2020 (post-COVID-19), at our main centre, the Royal Manchester Children’s Hospital, a total of 122 elective herniotomies were performed on 121 children, while 52 emergency herniotomies were performed on 51 children. Table 1 shows the distribution of cases between both time periods. Although we performed fewer elective herniotomies during the 2020 pandemic, children were not at more risk of undergoing an emergency herniotomy OR (95% CI) = 1.53 (0.79–2.9); *p* = 0.2.

**Table 1:** Number of herniotomies performed during the study period and number of children in whom a prior referral for an inguinal hernia was received or were seen in the outpatient department before elective or emergency herniotomy

Study period	Elective herniotomy (female to male)	Emergency herniotomy (female to male)	Total (female to male)	Emergency referral received, *n* (%)	Emergency seen in ^a^OPD, *n* (%)	Elective referral received, *n* (%)	Elective seen in OPD, *n* (%)
2019 April–Sept	76 (10: 66)	27 (5:22)	103 (15:88)	7 (25.9%)	2 (7.4%)	29 (38.1%)	66 (86.8%)
2020 April–Sept	46 (4: 42)	25 (5:20)	71 (9:62)	7 (28%)	2 (8%)	35 (76%)	33 (71.7%)
Total	122 (14: 108)	52 (10:42)	174 (24:150)	14 (27%)	4 (7.7%)	64 (52.5%)	99 (81%)

^a^*OPD* Outpatient department

The median (interquartile range [IQR]) age at elective surgery in 2019 was 7.3 months (3.8–18.8 months), compared to 2020 which was 5 months (3.3–21.5 months); *p* = 0.36. For emergency surgery, the median (IQR) age at surgery in 2019 was 7.9 months (2.65–53 months), compared to 2020 which was 2.8 months (2.1–13.7 months); *p* = 0.14. The difference noted in the age at emergency or elective herniotomy, before compared to during the pandemic, respectively, was not statistically significant.

**Fig. 1:**
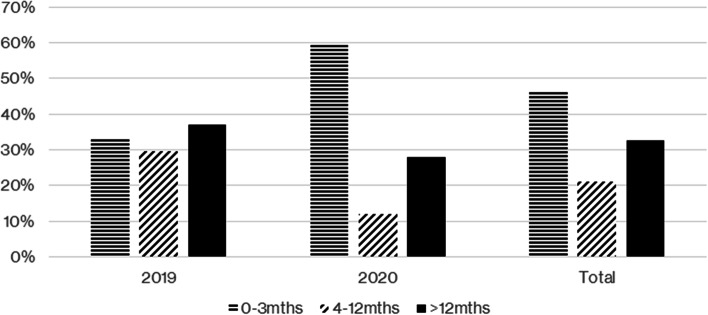
Age range distribution at emergency herniotomy

The child’s age was one of the criteria used to prioritise children for surgery. Figure 1 shows the age distribution of children that required emergency herniotomy. Overall, there was a higher percentage of infants aged 0 to 3 months that required emergency surgery. In 2019, slightly more children aged over 12 months required emergency surgery, and overall, this was the next more common (second commonest) age to suffer from an incarcerated hernia.

Table 1 shows the number of children in whom a referral for an inguinal hernia was received or were seen in the OPD prior to their surgery. There were a variety of reasons why children underwent elective herniotomies without a referral received or being seen in the OPD. These included some being seen and booked electively from the emergency department, some were booked from the ward (particularly the neonatal intensive care unit) and the others were seen as follow up for another condition (or a prior contralateral hernia) and a hernia diagnosed and then scheduled electively. There were also a few cases where the data on, when their referral was received, was unavailable. We discovered that only a small percentage of children that underwent emergency herniotomies were known to our service (referral received or seen in the OPD; Table 1).

Incarceration rates were defined as children seen and diagnosed as having an inguinal hernia in the OPD and while awaiting elective surgery present with a stuck hernia that requires emergency surgery. By this definition, our 2019 incarceration rate was 2.9% (2/68), compared to 2020 which was 5.7% (2/35); *p* = 0.6; thus, a relatively higher incarceration rate of was still noted during COVID-19, but not statistically significant.

For the children that a referral was received or were seen in OPD, Table 2 shows the duration of time they waited until they had elective surgery.

**Table 2:** Children’s wait time in days between referral received/seen in the outpatient department and elective surgery

Wait time in days	Median (days)	Interquartile range (days)
2019 referral to surgery	56	35–126
2019 ^a^OPD to surgery	73	37.5–129.25
2019 referral to OPD	13	8–18
2020 referral to surgery	59	26.5–196.5
2020 OPD to surgery	83	28–157
2020 referral to OPD	15	8.7–56.2

^a^OPD outpatient department

There was a slight increase in wait times during the 2020 pandemic; however, this was not statistically significant compared to 2019 referral received to surgery wait time (*p* = 0.61). Similarly, there was no significant difference when the 2019 vs 2020 OPD to surgery wait time and 2019 vs 2020 referral to OPD wait times were compared (*p* = 0.84 and *p* = 0.24, respectively).

## Discussion

The quick recognition for the need to continue delivering surgical care to children during the pandemic led to the development of several guidelines for safely delivering elective services [1, 4–6]. Based on these guidelines, our institution formulated a dynamic local policy. Three aspects were considered: OPD activity, scheduling process, and operating theatre procedures.

### OPD activity

We reinstituted “hot clinics” to see urgent patients which included inguinal hernia referrals in infants younger than 3 months old or any symptomatic child. We reduced the numbers of patients booked into these clinics to allow time to decontaminate the room between consultations and allow two-metre social distancing in the waiting area. We commenced virtual clinics (video and telephone clinics) to follow up new referrals and patients who were awaiting review or surgery and provide safety-netting advice on signs of hernia incarceration.

### Scheduling process and theatre procedures

Details of the scheduling process and theatre procedures can be found in the supplemental document, *New Pathway for Elective Admission of Children*. This guidance relied on the COVID-19 local/regional/national prevalence rates which were monitored [10] and categorised as green 0.5%, amber 0.5–2% and red greater than 2% prevalence.

By adhering to this policy, although our operating turnaround time between cases increased, thus reducing capacity, we successfully delivered elective surgery to children with various conditions.

Inguinal herniotomy is one of the more common procedures performed in children. An incarcerated inguinal hernia can have significant morbidity including bowel necrosis that requires laparotomy and bowel resection. Incarceration has been reported to be more likely the longer children wait to have surgical correction of their hernia [11]; however, some other authors have not corroborated this finding [12].

We discovered that during the pandemic, children did not wait much longer to get assessed compared to a similar time period before the pandemic. A recent systematic review [11] reported a median (range) wait time to surgery of 46 days (1–552 days), which compares favourably to our wait times, before and during the pandemic.

Furthermore, children were not more likely to undergo emergency herniotomy for incarceration during the pandemic. Median (range) incarceration rates for children awaiting herniotomy surgery has been reported as 8% (0–56%) [11]. We report less incarceration rates, before and during the pandemic.

It was interesting to note that most children that required emergency herniotomy were previously undiagnosed; however, this needs verification in larger and longer-term studies. These first presentations as incarcerations may be due to parents not recognising the problem, recognising it too late, or unable to access primary care in a timely manner. This suggests that more public health education could be done to prevent this. This may also support the notion and proposal, as per our findings, that when parents are seen and shown how to reduce the hernia, the children are then less likely to develop an incarcerated hernia while awaiting surgery [12].

The Royal College of Surgeons of England’s guidelines recommending prioritisation of hernia surgery in infants 0–3 months old [4] thus remain appropriate, because we found this age group more likely to undergo emergency herniotomy. Neonates, particularly those that were premature, have been reported to be at more at risk for incarceration [11, 13], but not confirmed by other reports [12]. By prioritising surgery in this age group, we prevented a rise in the incidence of incarcerated hernias. Children over 12 months appeared to be the next more common age group to develop incarceration; this finding requires more robust investigation to determine if their prioritisation timeframe should be reviewed during recovery of services from the pandemic and in the upcoming updates of the prioritisation guidance [14].

The limitations of this study include the unknown impact of delayed primary care referrals during the pandemic. Additionally, we did not analyse elective work at our secondary sites, most of which were cancelled at the start of the pandemic, but later reintroduced with the same guidelines. Surgeons may have also had a lower threshold to get hernias that presented to the emergency department during the pandemic (but were reduced without significant difficulty) done as an emergency, due to limited elective lists. Furthermore, some children were not brought to their first clinic appointment causing longer delays to being seen and some patients had surgery cancelled due to non-COVID-19-related sickness and so prolonging time from OPD to surgery. Finally, the data on children and guardians who became COVID-19-positive after coming in for elective surgery was unavailable; this would have contributed useful insight to the effectiveness of our strategy. Importantly, as this study was a single-centre study, the sample size did not allow establishing statistical significance for several points such as the difference in wait times and incarceration rates. This might need larger pooling of data from several centres and/or similar national level studies.

## Conclusions

By formulating and adhering to local guidelines developed from national guidance issued by the FSSA, RCPCH, and NICE, the COVID-19 pandemic did not result in children waiting significantly longer to be seen by a surgeon for a suspected inguinal hernia. As a result, we did not perform more emergency herniotomies for inguinal hernia incarcerations during the pandemic, and the majority of emergency herniotomies performed were first presentations. Improved public health education on childhood hernias and increased awareness of the need for prompt assessment and referral at primary care level will lead to even better outcomes. Finally, urgent prioritisation of hernias in infants 0–3 months old is appropriate because they were more likely to require emergency herniotomy. Such experience from our successes and shortcomings during this pandemic should be popularised and taken into consideration in the event of a force majeure requiring regimented pre-contemplated pathways and strenuous resource allocation.

## Supplementary Information

**Additional file 1.**

## Data Availability

The data and material for this study are available and stored confidentially.

## Abbreviations

COVID-19: Coronavirus Disease 2019
SARS-CoV-2: Severe Acute Respiratory Syndrome Coronavirus 2
OR: Odds Ratio
CI: Confidence Interval
IQR: Interquartile Range
OPD: Outpatient Department
FSSA: Federation of Surgical Specialty Associations
NICE: National Institute for Health and Care Excellence
RCPCH: Royal College of Paediatrics and Child Health
NHS: National Health Service
GIRFT: Getting It Right First Time (national programme)
