# Supplementary material for: Successful prioritisation of inguinal herniotomies in children during the COVID-19 pandemic to minimise emergency presentations
Source: Ann Pediatr Surg. 2023 May 1;19(1):20. doi: 10.1186/s43159-023-00243-1 (PMC10149152; doi:10.1186/s43159-023-00243-1)
Supplement: Supplementary file 1 — Additional file 1. [file 43159_2023_243_MOESM1_ESM.pdf]

Manchester University NHS Foundation Trust

New Pathway for Elective Admission of Children

Version 2.1, published 15th October 2020

Interpretation and summary of NICE guidance: COVID-19 rapid guidance: arranging planned care in hospitals and diagnostic services

Published 27 July 2020

www.nice.org.uk/guidance/ng179

National guidance for the recovery of elective surgery in children. RCPCH

Published: 27th July 2020, updated 15th October 2020

https://www.rcpch.ac.uk/resources/national-guidance-recovery-elective-surgery-children

Rachael Barber, Deputy Medical Director
Jai Sivaprakasam, Consultant Anaesthetist

1. INTRODUCTION

The purpose of this guideline is to help healthcare professionals deliver efficient planned care while minimising the risk of COVID-19 in the context of increasing or decreasing local prevalence. It also aims to help patients make decisions about their planned care.

It is for young people and children in hospitals and diagnostic settings. Planned care covers elective surgery (day surgery and inpatient stays), interventional procedures, diagnostics, imaging and planned medical admissions. It does not include services where people have ongoing outpatient and day-case procedures such as chemotherapy, radiotherapy and dialysis.

The document will apply to all paediatric patients within MFT regardless of their location/hospital of care (RMCH, WTWA, Dental/Eye Hospital).

This document has been updated to reflect NICE [1]and RCPCH[2] guidance (Sept 2020).

Core Principles

- The safety of children, their families and staff is paramount.
- Recommendations are to be equitable irrespective of socioeconomic status, ethnicity, or geographic location. No child should be left behind as a consequence of these recommendations
- Recommendations are evidence-based and may change as evidence and experience evolves
- Recommendations are to be responsive to the prevalence of COVID-19 in the community
- Guidance must link in with PHE and MFT Infection/Prevention Control guidance

2. BACKGROUND PRINCIPLES

The community prevalence of COVID-19 is currently low, with all areas having prevalence levels below 0.5% (correct 5 September 2020); in children the prevalence is lower than adults. The prevalence of COVID-19 in hospitals is significantly higher than that in the community, so by entering a hospital, a patient (and their parent) becomes at higher risk of infection. Currently, healthcare workers are more likely to contract COVID-19 from their colleagues rather than from patients or parents. However, if community prevalence increases significantly, the risk profile to patients and healthcare workers (HCW) shifts to the patients being more likely to infect the HCW.

There is an expectation that the prevalence of COVID-19 will remain low in the immediate future, but it is recognised that there may be local variation and that rates may increase in the winter months. PHE England and the Office for National Statistics (ONS) track week-on-week data to look at the prevalence of COVID-19 over time. PHE data systems are sufficiently robust to pick up these changes in growth and there is confidence that they will be able to alert local healthcare systems.

RCPCH recommends using a green/amber/red system based on accurate community prevalence rates to denote the risk level of performing elective surgery in children. For

---

[1] NG179: COVID-19 rapid guideline: arranging planned care in hospitals and diagnostic services. Pub 27 July 2020. *www.nice.org.uk/guidance/ng179*

[2] National guidance for the recovery of elective surgery in children. RCPCH 14th September 2020

RMCH which has a wide catchment area, the prevalence at the family's place of residence should be considered as well as regional and national prevalence.

Infection control recommendations should be amended in real time to reflect the level of risk. Although no changes in infection control measures are required when prevalence rises from low to moderate levels, there is an expectation that the situation is monitored closely in case the prevalence continues to rise to high rates. In this situation, there is expected to be a marked reduction in elective activity within hospitals during periods of high regional prevalence. It is important to note that by the first of September 2020, no area in local lockdown has exceeded the low prevalence threshold of 0.5%.

Public Health England will undertake a weekly review of regional prevalence levels and communicate this and the level of risk each Friday to the 10 Operational Delivery networks across England for them to cascade to hospitals. If the prevalence is significantly higher in a localized area (>2%) compared to the rest of the region, PHE will inform the individual ODN.

**Low**	Low levels of COVID-19 in community (prevalence < 0.5%)
**Moderate**	Low to moderate levels of COVID-19 in community (prevalence ?0.5% but <2%)
**High**	Moderate to high levels of COVID-19 in community (prevalence ?2%)

In addition to regional prevalence, it is recommended that children are stratified using a red/amber/green system in relation to PPE use linking in with the revised PHE recommendations on infection prevention control. Careful consideration should be taken about performing any elective surgery on children who fall into red or amber categories.

Red/Amber/Green Pathways for Elective Surgery for Children

	High risk COVID-19 pathway - Red	Medium risk COVID-19 pathway - Amber	Low risk COVID-19 pathway - Green
During Low and Medium Prevalence of COVID-19	a)Symptomatic or suspected COVID-19 individuals including those with a history of contact with a case<br><br>b) Confirmed COVID-19 on testing<br><br>c) Symptomatic individuals who decline testing	a) Asymptomatic for COVID-19 with no known recent contact but no test result available	a) No symptoms or known recent contact with COVID-19<br>**AND**<br>1 negative COVID-19 test within 72 hours<br><br>**OR**<br><br>b)Have recovered from COVID-19 with at least 3 consecutive days without fever or respiratory symptoms and a negative test
During High Prevalence of COVID-19	a) Symptomatic or suspected COVID-19 individuals including those with a history of contact with a case<br><br>b) Confirmed COVID-19 on testing<br><br>c) Symptomatic individuals who decline testing	a)Asymptomatic for COVID-19 with no known recent contact but no test result available<br><br>b)Single negative swab prior to treatment<br><br>c)Parent/carer and/or child have not shielded/isolated prior to treatment	a) No symptoms or known recent contact with COVID-19<br>**AND**<br>1 negative COVID-19 test within 72 hours before treatment (preferably a maximum of 24 hours before)<br><br>**OR**<br><br>b)Have recovered from COVID-19 with at least 3 consecutive days without fever or respiratory symptoms and a negative test

3. THEATRE LIST PLANNING

Management of the theatre waiting list will be determined by multiple factors including the clinical urgency of the procedure, changes in the clinical condition which have occurred between listing of a case and the time of operation, pre-operative assessment and (non-COVID) investigations which are required and taking consent. This is because the clinical indications for some procedures may have changed as a result if the positive impact of lockdown on children's health, e.g. reduced upper respiratory infections. In addition, adjustments in the planning of the theatre list may be required to consider additional protective measures in theatre, primarily the time required for air changes after airway manipulation.

Guidance on the recovery of surgical services and on the clinical urgency of the procedure has been published by the Royal College of Surgeons of England and identifies the following categories[3]

**Priority level 1a**	Emergency - operation needed within 24 hours
**Priority level 1b**	Urgent - operation needed within 72 hours
**Priority level 2**	Surgery that can be deferred for up to 4 weeks
**Priority level 3**	Surgery that can be delayed for up to 3 months
**Priority level 4**	Surgery that can be delayed for more than 3 months

During periods of low and moderate prevalence cases of category 1, 2, 3 and 4 urgency can be undertaken. During high periods every attempt should be made to continue elective surgery, but local providers should risk assess this and match capacity to service demands/staff availability.

4. PRE-ADMISSION

4.1. PRE-ASSESSMENT, INFORMATION FOR PARENTS AND MANAGING EXPECTATIONS

Pre-admission assessment of children undergoing surgery or admission is required. There are two key aims:

- COVID-19 risk assessment
- Pre-operative assessment

For patients being admitted for investigations/observations that don't involve a general anaesthetic, only the COVID-19 risk assessment pathway needs to be used.

COVID-19 Risk Assessment

All children and household members should undergo pre-admission virtual/telephone screening 24-72 hours pre-operatively. This should specifically ask about symptoms suggestive of COVID-19 infection. Please see Appendix 2 for RMCH/MCS proposed questionnaire. If symptoms are present in either the child or household members, advice should be given according to Government guidelines about COVID-19 testing and self-isolation and the admission/procedure should be delayed until a later date.

---

[3] Royal College of Surgeons. Clinical guide to surgical prioritisation during the coronavirus pandemic 2020 www.rcseng.ac.uk/coronavirus/surgical-prioritisation-guidance/

If the condition for which admission/surgery is required does not allow a delay, a discussion about the decision making around this should occur within a multidisciplinary team and the family. The patient and family members should be treated according to the COVID-19 positive patient pathway if the procedure goes ahead. If the child has mild coryzal symptoms and is otherwise well a pre-operative SARS-CoV-2 swab could be performed and if negative the child can continue to admission.

Screening of the child and household members for new symptoms of COVID-19 should be performed again at the time of admission. If a child develops mild coryzal symptoms after the pre-operative screening and have had a negative SARS-CoV-2 swab within 72 hours, a rapid swab could be performed to determine whether they have COVID-19 or another mild influenza like illness. If negative and the anesthetist is happy to proceed, it is acceptable for the child to follow the “green” pathway during times of low or moderate prevalence. If a child develops significant symptoms OR a household member has developed symptoms, the procedure should be delayed until a later date allows, and advice given according to Government guidelines about COVID-19 testing and self- isolation. If the condition for which admission/surgery is required does not allow a delay, a discussion about the decision making around this should occur within a multidisciplinary team and the family.

All families should receive or have access to guidance and advice about the infection control processes associated with elective admissions/procedures considering COVID-19. For RCPCH examples see Appendix 3

Pre-Operative Assessment

All non-emergency surgical patients should be assessed by a pre-assessment nurse specialist either by telephone or video platform.

Face to face assessment should be reserved for patients where adequate assessment is not possible by above methods.

The historic pathways for any concerns will still apply with respect to:

- Notes review
- Consultant anaesthetist review (initially by telephone)
- Pre-optimisation and further investigations

For detailed timeline and pre-admission screening tool see appendix 1 and 2.

5. PRE-OPERATIVE ISOLATION

RCPCH does not recommend pre-operative isolation as routine practice for children prior to admission.

As part of the pre-operative assessment for children requiring Paediatric Critical Care post-operatively, isolation may be recommended at the clinical teams’ discretion. This is not part of RCPCH guidance, but may be appropriate for very high risk surgery.

6. PRE-ADMISSION SARS-COV2 TESTING

Reverse transcriptase polymerase chain reaction (RT-PCR) performed on a single swab of throat then nose is the recommended test for diagnosing acute COVID-19. It has a high analytical sensitivity and can detect low levels of SARS-CoV-2 with good reproducibility.

For patients with a tracheostomy, tracheal swabs will have a higher yield of positivity and should be taken.

A single pre-operative swab should be taken a maximum of 72 hours before admission and preferably as close to the time of surgery as possible. Ideally this would occur on the day of surgery, although this is not feasible with current limitations to point of care testing. The rationale for performing this test is to reassure theatre staff, although there is still a risk of the patient developing COVID-19 during their inpatient stay.

During periods of high prevalence (>2%) pre-operative swabbing in the 24 hours prior to surgery is recommended. If this is not achievable testing within a hospital setting within 72 hours should be performed. This is to ensure the rapid turnaround of results to enable swabbing to be performed as close to the time of procedure as possible

For patients travelling long distances, local testing should be arranged if possible.

As part of the pre-operative assessment for children requiring Paediatric Critical Care post-operatively, two pre-operative swabs may be considered at the clinical teams' discretion.

For inpatients requiring surgery, the RMCH MCS SOP for COVID-19 Testing for Inpatient Wards should be used.

7. PRE-ADMISSION CONSENT

There are several issues to consider with regard to pre-operative consent during this time. The Royal College of Surgeons has published guidelines on this matter[4].

To summarise:

- It is a recognised standard of care that consent for elective surgery should not happen on the day of surgery. The RCS has stated very clearly that a minimum period of 48 hours should elapse between taking consent and surgery. A recent medicolegal case resulted in a judgment whereby the judge effectively ruled that consent taken on the day of surgery was not valid commenting that informed consent required 'adequate time and space'. The RCS state in their guidelines '*...when it comes to the consent process, the same principles and requirements should apply as set out by the GMC and The Royal College of Surgeons of England, regardless of whether the conversation takes place face-to-face or via phone or video.*'

- Children and their parents/carers should have the opportunity to discuss the procedure prior to elective admission and have access to written information.

[4] RCS. Consent to Treatment, While COIVD-19 is Prevalent in Society. www.rcseng.ac.uk/coronavirus/recovery-of-surgical-services/tool-5/] and RCS Good Surgical Practice - 3.5.1 Consent

- Departments should make their own arrangements, within the constraints placed upon them by Covid-19, to ensure that consent is taken in a way which allows parents and carers to weigh the options for treatment and the risks involved adequately before proceeding with surgery. This could be via remote consultation and the posting of consent forms or with a suitably timed face to face consultation.

- Wherever possible, written information sheets should be supplied to children, parents and carers.

- Parents and carers should be warned of the risks of contracting Covid-19 and the potential implications of this for children undergoing elective surgery. The evidence base on which to base this conversation is evolving and, inevitably, these conversations will have to reflect our current state of knowledge in this area. The RMCH covid-19 consent form should be used to inform families of this risk.

8. PERI-OPERATIVE CONSIDERATIONS

Number of parents/carers per child:

MFT visitor policy should be followed. The current version (V3 24th July 2020) advises the following for children's wards:

- One parent/named family member may be resident with the child. Resident parents must comply with safety measures at all times.
- The needs of children with a learning disability and/or autism must be assessed on the day of admission and daily thereafter and an individual visiting plan must be established to meet their needs.
- If a child's admission exceeds 7 days, one additional named visitor may visit at a pre-arranged time for a defined time period, if this is assessed as necessary and can be safely accommodated.
- Visiting times are not restricted but this may be changed by the COVID-19 Strategic Group in response to the assessed level of risk at any time

Ideally the resident-carer should not have a co-morbidity which requires shielding; this should be discussed at the pre-operative assessment.

Face-coverings for children and adults attending hospital for elective procedures

All resident carers should wear a face covering while in hospital. Children over the age of 12 years should be encouraged to wear face coverings on corridors/in public areas if tolerated. MFT Visitor and IPC policies should be followed.

Place of Admission

Elective admissions who have completed all aspects of pre-admission COVID screening and have a negative COVID-19 swab can be admitted to low-risk areas within RMCH/MCS.

Children that have not completed the above should be managed as amber, medium risk patients as per current infection control guideliens. Where possible, cubicles should be prioritised for children and accompanying parents with comorbidities for whom shielding is required.

Healthcare Workers

All healthcare workers should wear appropriate PPE as per PHE guidance for contact with patient and their family during the hospital stay.

9. INTRA-OPERATIVE CONSIDERATIONS

Please see General Anaesthesia process for patients in RMCH and MCS theatres during period of sustained transmission of COVID-19 version 1 for current information.

Minimum PPE requirements in theatre according to regional prevalence and screening pathway

	Low and moderate regional prevalence (<2%)			High regional prevalence (?2%)		
	Green	Amber	Red	Green	Amber	Red
AGPs	FRSM II Apron Gloves	Eye protection FFP3 / Hood Fluid-repellant gown Gloves	Eye protection FFP3 / Hood Fluid-repellant gown Gloves	Eye protection FFP3 / Hood Fluid-repellant gown Gloves	Eye protection FFP3 / Hood Fluid-repellant gown Gloves	Eye protection FFP3 / Hood Fluid-repellant gown Gloves
Non-AGPs	FRSM II Apron Gloves	FRSM II Apron Gloves	FRSM II Apron Gloves	FRSM II Apron Gloves	FRSM II Apron Gloves	FRSM II Apron Gloves
Cleaning	Standard cleaning	Enhanced Cleaning	Enhanced Cleaning	Enhanced Cleaning	Enhanced Cleaning	Enhanced Cleaning
Air changes after AGP	None	5 air changes	5 air changes	5 air changes	5 air changes	5 air changes
Extubation in theatre	FRSM II Apron Gloves	Eye protection FFP3 / Hood Fluid-repellant gown Gloves	Eye protection FFP3 / Hood Fluid-repellant gown Gloves	Eye protection FFP3 / Hood Fluid-repellant gown Gloves	Eye protection FFP3 / Hood Fluid-repellant gown Gloves	Eye protection FFP3 / Hood Fluid-repellant gown Gloves
LMA removal In theatre or recovery	FRSM II Apron Gloves	Eye protection FFP3 / Hood Fluid-repellant gown Gloves	Eye protection FFP3 / Hood Fluid-repellant gown Gloves	Eye protection FFP3 / Hood Fluid-repellant gown Gloves	Eye protection FFP3 / Hood Fluid-repellant gown Gloves	Eye protection FFP3 / Hood Fluid-repellant gown Gloves
Oropharyngeal removal In theatre or recovery	FRSM II Apron Gloves	FRSM II Apron Gloves	FRSM II Apron Gloves	FRSM II Apron Gloves	FRSM II Apron Gloves	FRSM II Apron Gloves

10. POST-OPERATIVE AND DISCHARGE CONSIDERATIONS

Normal post-operative observations and care pathways should be followed.

Consistent messaging regarding hand washing, social distancing and face coverings should be maintained throughout the hospital to reinforce that these infection prevention and control measures are the most effective way of reducing the transmission of COVID-19.

Rapid discharge after day case procedures should be supported and encouraged.

Children and resident-carer do not need to self-isolate after discharge from hospital unless they have been diagnosed with COVID-19.

Virtual or telephone clinics should be supported to improve access to healthcare for all patients and to reduce the number of children who need to return to the hospital for outpatient review

11. MONITORING

Surveillance of nosocomial outbreaks and monitoring of outcomes of children after surgery should be undertaken.

If a child undergoes an elective procedure within 14 days of a positive swab for SARS-CoV-2, it is recommended they are reported to the www.covidinchildren.co.uk database to enable monitoring of outcomes.

12. APPENDIX 1 – PRE-ADMISSION TIMELINE FOR SCHEDULERS TO ENSURE GREEN PATIENTS

D - 28

Final scheduling decision and list identified

↓

D -14

Telephone Pre-assessment by pre op nurse	Share patient/ family information leaflets including COVID-19 related advice

↓

D -7

Anaesthetic virtual review/ MDT input if needed	Review COVID-19 prevalence and follow appropriate screening pathway

↓

D - 5-7

Bloods/ investigations/ face to face assessment if required (one-stop shop)

↓

D -2-3

Covid19 swab and pre-admission screening questionairre

↓

D -1

Chase swabs and confirm negative

↓

D 0

Pre-admission screening at door	Admit to ward

13. APPENDIX 2 – ELECTIVE ADMISSION COVID-19 SCREENING TOOL

Symptom Questions	
1. Has the child had temperature of more than 37.8 C, within last 7 days?	Yes/ No
2. Has the child had a **new-onset** of a cough or breathlessness, within the last 7 days?	Yes/No
3. Has the child had any loss of smell or taste in the last 7 days?	Yes/No
3. Does the child have any of the following symptoms (**new onset**) that have occurred in the last 7 days?	
a. Abdominal symptoms (pain, diarrhoea, vomiting)	Yes/ No
b. Sore throat	Yes/ No
c. Rash, redness of eyes, swollen hands/ feet	Yes/ No
d. Joint, muscle ache or flu-like symptoms	Yes/ No
4. a. Has the child or anyone in the family had any contact with a Covid-19 positive patient in the last 14 days?<br><br>b. Has the child or anyone living with the child, asked by NHS Test and Trace to self-isolate?	Yes/ No
5. Has anyone in the household suffered from covid 19 symptoms (fever, cough, breathlessness) and/or been self-isolating in the last 14 days?	Yes/ No

	Suspicion of Covid-19
Yes to 1 or more questions	Moderate to High<br><br>Escalate to medical or senior nursing team before proceeding
No to all the questions	Low to very-low

Acting on a Positive Swab

Relevant medical team should be informed

The decision to proceed with a positive swab should be reviewed and authorized by the CSU Leads for Anaesthesia and Surgery

PHE isolation advice should be provided and contact tracing initiated as per Trust and PHE guidance

14 day recovery period before re-swabbing

14. PATIENT AND PARENT INFORMATION LEAFLETS

Information for parents and carers about planned admissions for surgery/investigation under general anaesthesia

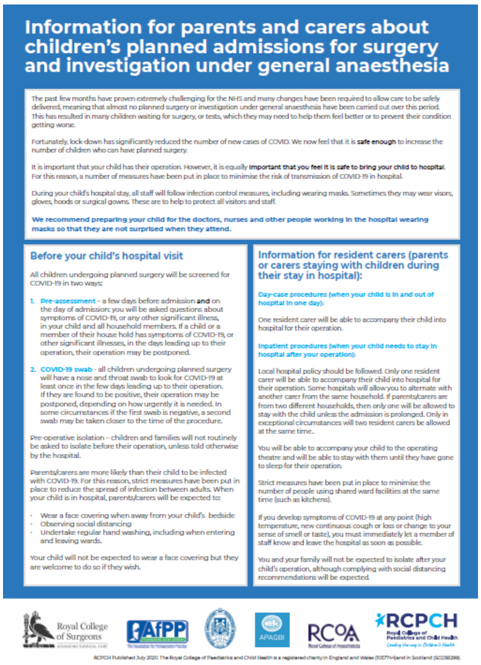

Information for parents and carers about children's planned admissions for surgery and investigation under general anaesthesia

The past few months have proven extremely challenging for the NHS and many changes have been required to allow care to be safely delivered, meaning that almost no planned surgery or investigation under general anaesthesia have been carried out over this period. This has resulted in many children waiting for surgery, or tests, which they may need to help them feel better or to prevent their condition getting worse.

Fortunately, lock-down has significantly reduced the number of new cases of COVID. We now feel that it is **safe enough** to increase the number of children who can have planned surgery.

It is important that your child has their operation. However, it is equally **important that you feel it is safe to bring your child to hospital**. For this reason, a number of measures have been put in place to minimise the risk of transmission of COVID-19 in hospital.

During your child's hospital stay, all staff will follow infection control measures, including wearing masks. Sometimes they may wear visors, gloves, hoods or surgical gowns. These are to help to protect all visitors and staff.

We recommend preparing your child for the doctors, nurses and other people working in the hospital wearing masks so that they are not surprised when they attend.

Before your child's hospital visit

All children undergoing planned surgery will be screened for COVID-19 in two ways:

1. Pre-assessment – a few days before admission **and** on the day of admission: you will be asked questions about symptoms of COVID-19, or any other significant illness, in your child and all household members. If a child or a member of their house hold has symptoms of COVID-19, or other significant illnesses, in the days leading up to their operation, their operation may be postponed.
2. COVID-19 swab - all children undergoing planned surgery will have a nose and throat swab to look for COVID-19 at least once in the few days leading up to their operation. If they are found to be positive, their operation may be postponed, depending on how urgently it is needed. In some circumstances if the first swab is negative, a second swab may be taken closer to the time of the procedure.

Pre-operative isolation – children and families will not routinely be asked to isolate before their operation, unless told otherwise by the hospital.

Parents/carers are more likely than their child to be infected with COVID-19. For this reason, strict measures have been put in place to reduce the spread of infection between adults. When your child is in hospital, parents/carers will be expected to:

- Wear a face covering when away from your child's bedside
- Observing social distancing
- Undertake regular hand washing, including when entering and leaving wards.

Your child will not be expected to wear a face covering but they are welcome to do so if they wish.

Information for resident carers (parents or carers staying with children during their stay in hospital):

Day-case procedures (when your child is in and out of hospital in one day):

One resident carer will be able to accompany their child into hospital for their operation.

Inpatient procedures (when your child needs to stay in hospital after your operation):

Local hospital policy should be followed. Only one resident carer will be able to accompany their child into hospital for their operation. Some hospitals will allow you to alternate with another carer from the same household. If parents/carers are from two different households, then only one will be allowed to stay with the child unless the admission is prolonged. Only in exceptional circumstances will two resident carers be allowed at the same time..

You will be able to accompany your child to the operating theatre and will be able to stay with them until they have gone to sleep for their operation.

Strict measures have been put in place to minimise the number of people using shared ward facilities at the same time (such as kitchens).

If you develop symptoms of COVID-19 at any point (high temperature, new continuous cough or loss or change to your sense of smell or taste), you must immediately let a member of staff know and leave the hospital as soon as possible.

You and your family will not be expected to isolate after your child's operation, although complying with social distancing recommendations will be expected.

RCPCH Published July 2020. The Royal College of Paediatrics and Child Health is a registered charity in England and Wales (1057744)and in Scotland (SC038299).

Information for young people about planned admission to hospital

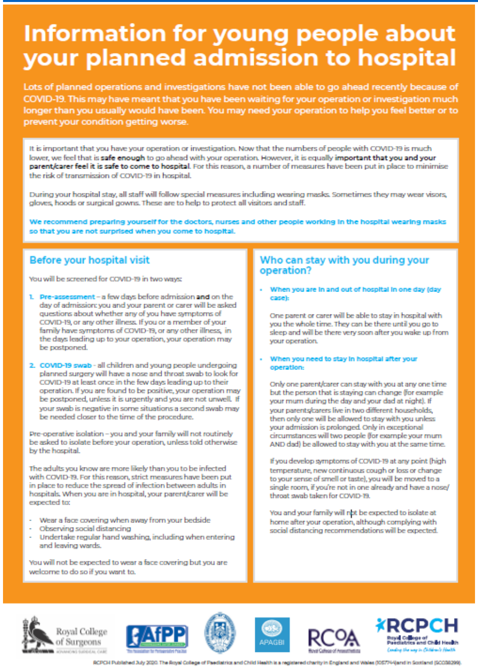

Information for young people about your planned admission to hospital

Lots of planned operations and investigations have not been able to go ahead recently because of COVID-19. This may have meant that you have been waiting for your operation or investigation much longer than you usually would have been. You may need your operation to help you feel better or to prevent your condition getting worse.

It is important that you have your operation or investigation. Now that the numbers of people with COVID-19 is much lower, we feel that is **safe enough** to go ahead with your operation. However, it is equally **important that you and your parent/carer feel it is safe to come to hospital**. For this reason, a number of measures have been put in place to minimise the risk of transmission of COVID-19 in hospital.

During your hospital stay, all staff will follow special measures including wearing masks. Sometimes they may wear visors, gloves, hoods or surgical gowns. These are to help to protect all visitors and staff.

We recommend preparing yourself for the doctors, nurses and other people working in the hospital wearing masks so that you are not surprised when you come to hospital.

Before your hospital visit

You will be screened for COVID-19 in two ways:

1. Pre-assessment – a few days before admission **and** on the day of admission: you and your parent or carer will be asked questions about whether any of you have symptoms of COVID-19, or any other illness. If you or a member of your family have symptoms of COVID-19, or any other illness, in the days leading up to your operation, your operation may be postponed.
2. COVID-19 swab - all children and young people undergoing planned surgery will have a nose and throat swab to look for COVID-19 at least once in the few days leading up to their operation. If you are found to be positive, your operation may be postponed, unless it is urgently and you are not unwell. If your swab is negative in some situations a second swab may be needed closer to the time of the procedure.

Pre-operative isolation – you and your family will not routinely be asked to isolate before your operation, unless told otherwise by the hospital.

The adults you know are more likely than you to be infected with COVID-19. For this reason, strict measures have been put in place to reduce the spread of infection between adults in hospitals. When you are in hospital, your parent/carer will be expected to:

- Wear a face covering when away from your bedside
- Observing social distancing
- Undertake regular hand washing, including when entering and leaving wards.

You will not be expected to wear a face covering but you are welcome to do so if you want to.

Who can stay with you during your operation?

- **When you are in and out of hospital in one day (day case):**

  One parent or carer will be able to stay in hospital with you the whole time. They can be there until you go to sleep and will be there very soon after you wake up from your operation.

- **When you need to stay in hospital after your operation:**

  Only one parent/carer can stay with you at any one time but the person that is staying can change (for example your mum during the day and your dad at night). If your parents/carers live in two different households, then only one will be allowed to stay with you unless your admission is prolonged. Only in exceptional circumstances will two people (for example your mum AND dad) be allowed to stay with you at the same time.

  If you develop symptoms of COVID-19 at any point (high temperature, new continuous cough or loss or change to your sense of smell or taste), you will be moved to a single room, if you're not in one already and have a nose/ throat swab taken for COVID-19.

  You and your family will not be expected to isolate at home after your operation, although complying with social distancing recommendations will be expected.

RCPCH Published July 2020. The Royal College of Paediatrics and Child Health is a registered charity in England and Wales (1057744)and in Scotland (SC038299).

Information for children- my Operation and Coronavirus
